# Machine learning models explanations as interpretations of evidence: a theoretical framework of explainability and its implications on high-stakes biomedical decision-making

**DOI:** 10.1186/s12874-025-02703-1

**Published:** 2025-12-31

**Authors:** Matteo Rizzo, Alberto Veneri, Matteo Marcuzzo, Alessandro Zangari, Andrea Albarelli, Claudio Lucchese, Marco Salvatore Nobile, Cristina Conati

**Affiliations:** 1 Department of Environmental Sciences, Informatics and Statistics, Ca’ Foscari University, Dorsoduro 3246, 30123 Venice, Italy; 2 Institute of Information Science and Technologies (ISTI), National Research Council of Italy (CNR), via G. Moruzzi 1, 56124 Pisa, Italy; 3 Department of Computer Science, University of British Columbia, ICICS/CS Building 201-2366 Main Mall, V6T 1Z4 Vancouver, British Columbia Canada

**Keywords:** Explainability, Interpretability, Machine learning, Biomedicine

## Abstract

Explainable Artificial Intelligence, or XAI, is a vibrant research topic in the artificial intelligence community. It is raising growing interest across methods and domains, especially those involving high-stakes decision-making, such as the biomedical sector. Much has been written about the subject, yet XAI still lacks shared terminology and a framework capable of providing structural soundness to explanations, a crucial need for decisions that impact healthcare. In our work, we address these issues by proposing a novel definition of explanation that synthesizes insights from the existing literature. We recognize that explanations are not atomic, but rather the combination of evidence stemming from the model and its input-output mapping, along with the human interpretation of this evidence. Furthermore, we fit explanations into the properties of faithfulness (i.e., the explanation is an accurate description of the model’s inner workings and decision-making process) and plausibility (i.e., how much the explanation seems convincing to the user). Our theoretical framework simplifies the operationalization of these properties and provides new insights into common explanation methods that we analyze through case studies. We explore the impact of our framework in the sensitive domain of biomedicine, where XAI can play a central role in generating trust by balancing faithfulness and plausibility.

## Introduction

The advent of Deep-Learning (DL) increased the accuracy bar of Machine Learning (ML) models for countless tasks and domains. Riding the enthusiasm around such stunning results, DL models have been deployed even in high-stakes decision-making environments, but not without criticism [1–3]. Biomedicine is one of these environments. It requires high predictive accuracy and an *explanation* of why that prediction was made. The need for explanations initiated a discussion around the explainability of DL models, which are known to be “black boxes”. In other words, their inner workings are hard for humans to understand. Who should be accountable for a model-based decision and how a model came to a certain prediction are just some of the questions that drive research on explaining ML models.

This is particularly relevant, for instance, in the biomedical field, where human lives are at stake, and understanding the reasoning behind a model’s predictions is essential to guarantee safety and avoid costly errors [4]. Furthermore, explaining how a model arrives at a certain conclusion can increase understanding of the underlying biological mechanisms, enabling more informed support for decision-making by clinicians and researchers, as well as facilitating knowledge discovery.

With the first recent attempts of the legislative machinery to make explanations for automatic decisions a user’s right [5], the pressure on generating explanations for the ML model’s behaviors increased even more. Despite the endeavor of the eXplainable Artificial Intelligence (XAI) community to develop either models that are explainable by design [6–8] and methods to explain existing black-box models [9–11], the way to DL explainability is paved with results that are mostly preliminary and do not fit industry scale (e.g., [12–15]). Most notably, it is challenging to relate different pieces of research due to a lack of common theoretical grounds that can support and guide the discussion. In particular, we identify a gap in the literature regarding foundational issues, such as a shared definition of the term “explanation” and the role of users in the design and deployment of explainability for complex ML models. The XAI community suffers from the paucity of common terminology, with only a few attempts to establish one, focusing more on the distinction among the terms “interpretable”, “explainable”, and “transparent” rather than the inner structure and meaning of an explanation (e.g., [16–18]). Similarly, a lack of an outline of the main theoretical components of the discussion around explainability disperses research. At the same time, the current literature struggles to provide the involved stakeholders with principled analytical tools to operate on black-box models. This trend has been detected in the delicate field of biomedicine and addressed with context-specific guidelines [19].

Numerous works have discussed the application of explanation techniques to black-box models in clinical scenarios, either analyzing general approaches, such as [20] and [21], or investigating specific types of models, such as argumentation approaches [22]. However, to our knowledge, no works in the literature have presented a general and formal characterization of what an explanation technique for an automated system is and how to characterize the process of creating it.

In this work, we propose a simple, general, and effective theoretical framework that outlines the core components of the explainability machinery and lays out grounds for a more coherent debate on how to explain the decisions of ML models. Such a framework is not meant to be set in stone, but rather used as a common reference among researchers and iteratively improved to accommodate more sophisticated explainability methods and strategies. We aim to provide shared jargon and formal definitions to inform and standardize discussions around crucial topics in XAI. The core of the proposed theoretical framework is a novel definition of ’explanation’ that draws from existing literature in sociology and philosophy, yet is also easy to operationalize when analyzing a specific approach to explaining the predictions made by a model. We conceive an explanation as the interaction of two decoupled components, namely *evidence* and its *interpretation*. “Evidence” is any information stemming from a ML model. At the same time, an ’interpretation’ refers to the semantic meaning that human stakeholders attribute to the evidence, enabling them to make sense of the model’s inner workings. Thus, an “explanation” results from applying an “interpretation” (i.e., the meaning) to some evidence (i.e., factual data).

We relate these definitions to crucial properties of explanations, especially *faithfulness* and *plausibility*. In their seminal paper on the topic, Jacovi & Goldberg define faithfulness as “the accurate representation of the causal chain of decision-making processes in a model” [23]. We argue that faithfulness relates to the elements of the proposed theoretical framework in different ways, as it ensures that the interpretation of the evidence is faithful to how the model utilizes it within its inner reasoning. A property orthogonal to faithfulness is plausibility, namely “the degree to which some explanation is aligned with the user’s understanding of the model’s decision process” [23]. A follow-up work by Jacovi & Goldberg addresses plausibility as the “property of an explanation of being convincing towards the model prediction, regardless of whether the model was correct or whether the interpretation is faithful” [24]. We relate plausibility to faithfulness and highlight the need for faithfulness to be embedded in explainability methods and strategies, while acknowledging plausibility as an important, yet not indispensable, property. This is particularly true in the biomedical field, which is a high-stakes environment where it is crucial for the explanation to accurately portray the model’s decision-making process.

As case studies, we delve into the evaluation of faithfulness of some popular DL explanation tools and strategies, such as “attention” [11, 25], Gradient-weighted Class Activation Mapping (Grad-CAM) [26], and SHapley Additive exPlanations (SHAP) [10]. In addition, we look at the faithfulness of models traditionally considered intrinsically interpretable (a notion we distance ourselves from), such as a *linear regressor* and models based on *fuzzy logic*.

## Designing explainability

Research in XAI seizes the problem of explaining models for decision-making from multiple perspectives. First, most existing literature uses the terms “interpretable” and “explainable” interchangeably, while some have highlighted the semantic nuance that distinguishes the two words [27, 28]. Among those who distinguish the two terms, “interpretable” is often associated with the condition of some model’s inner workings, or part of them, *to be* human-understandable. On the other hand, in many cases, the term “explainable” refers to the condition of a model’s functioning *being made* human-understandable. We offer a third perspective on this issue.

Firstly, we align with the policy of distinguishing between the meanings of the words “interpretable” and “explainable”. We argue that the term *explainable* (and, by extension, *explainability*) is more suited than the term *interpretable* (similarly, by extension, *interpretability*) to describe the property of a model for which effort is made to generate human-understandable clarifications of its decision-making process. The definition of *explanation* is thus crucial and will be discussed extensively in Defining explanations section.

Secondly, we attribute a new semantic meaning to the term “interpretation”. Our claim follows three rationales: *(i)* the verb “to interpret” means “explaining something” but also “understanding something as having a particular meaning” [29]. The first definition can be used explicitly by employing the verb “to explain” directly, while the second definition better suits how we utilize the term within our proposed theoretical framework; *(ii)* the term *interpretation* is used within our proposed framework with a precise meaning that deviates from the current literature and that we deem more accurate (see Definitions of the components of the framework section). We deem an “interpretation” the semantic that is associated with some quantitative evidence coming from the model and the input-output relationship; *(iii)* we argue against grouping models into *inherently interpretable* and *post-hoc explainable*, which naturally requires a different nuance of meaning between the terms “interpretable” and “explainable”, rooted in untamed literature. We now propose our justification for why this holds.

Recently, Molnar has defined “intrinsic interpretability” as a property of ML models that are considered entirely understandable due to their simple structure (e.g., short decision trees or sparse linear models), while “post hoc explainability” as the need for some models to apply interpretation methods after training [30]. Although principled, we drop this hard distinction by claiming all models embed a certain degree of explainability. Although, to our knowledge, no metric has precisely quantified explainability yet, we can assert that it depends on multiple factors. In particular, a model is as explainable as the explanations proposed to the user to justify a specific prediction are effective. Thus, bringing humans into the explainability design loop is critical to deploying explainable models.

Consequently, there are models for which it is easier to design explanations (i.e., the so-called *white-box* models, such as linear regression, decision trees, rule-based systems, etc.) and models for which the same process is more difficult (i.e., the so-called *black-box* models, e.g., artificial neural networks). The notion of difficulty here is defined by the inner complexity of the model, which relates to the amount of cognitive load the user can sustain when trying to understand its inner workings. We highlight that the degree of explainability progresses along a continuum from black-box to white-box models, without clear-cut thresholds. Nevertheless, in Case studies: framing common explainability strategies section, we show that explanations for both white-box and black-box models fit our proposed framework. Thus, they can both be structured homogeneously and be more deeply understood by leveraging theoretical tools.

Most importantly, we advocate for explainability design as a crucial part of Artificial Intelligence (AI) software development. We endorse Chazette et al., claiming that explainability should be considered a non-functional requirement in the software design process [31]. Thus, explanations for any ML models (and, especially, for DL models) should be accounted for within the initial design of an AI-powered application. Even the most accurate black-box model should not be deployed without an explanation mechanism backing it up, as we cannot be certain whether it has learned to discriminate between meaningful and incorrect features. A classic example is a dog image classification model that learns to detect huskies due to the snowy setting, rather than the features of the animal itself, inadvertently deceiving the users [9]. A design-oriented approach to AI development should involve taking humans into the loop, thereby fostering a human-centered AI that is more intelligible by design and is expected to increase trust in end-users [32].

### Explaining the model vs explaining the data (the world)

In ML discourse, the nuanced differentiation between explaining the mechanics of ML models and elucidating the datasets they process underscores a multifaceted challenge. This distinction is pivotal for achieving technical clarity and fostering a comprehensive understanding spanning the socio-technical landscape within these models.

The endeavor to explain datasets, thus unraveling the complexities of the real-world phenomena they encapsulate, transcends technical analysis, delving into the socio-economic, cultural, and ethical dimensions that underpin data generation and collection methodologies. A critical aspect of data explainability is acknowledging the constructed nature of datasets. Data are not neutral or objective; they are imbued with the biases and perspectives of those who collect, curate, and process them. This recognition is essential for addressing ML application representation, bias, and fairness issues. Exploring the nuances of data provenance and bias necessitates critically examining how data are gathered and the assumptions underlying their collection. Datasets often reflect historical inequalities, systemic biases, and societal norms that can perpetuate discrimination and inequity when used uncritically in ML models [33]. Addressing these challenges requires a conscientious approach that encompasses ethical considerations, transparency in data curation, and active efforts to mitigate bias.

The relationship between model and data explainability is not merely sequential but deeply interwoven. While techniques such as LIME and SHAP offer insights into the operational aspects of model predictions, they often fall short of providing a comprehensive understanding of the underlying data narratives [9, 10]. To overcome the limitations of these approaches, there is a pressing need for frameworks that clarify the mechanisms of model decisions and interrogate the origins, biases, and implications of the data they analyze. This integrative perspective emphasizes the importance of contextual knowledge and ethical scrutiny in the development and deployment of ML systems. It advocates for a multidisciplinary approach that marries technical expertise with insights from social sciences, ethics, and domain-specific knowledge. Such a synthesis is crucial for ensuring that ML technologies are explainable and aligned with societal values.

The dichotomy between explaining models and data underscores a broader imperative in ML: cultivating a holistic understanding that bridges technical intricacies with ethical and societal considerations. Achieving balance is indispensable for developing ML systems that are both intelligible, responsible, and beneficial. As the field evolves, fostering interdisciplinary collaboration and prioritizing ethical considerations will be paramount in navigating the complexities of this landscape.

## Characterising the inference process of an ML model

**Fig. 1:**
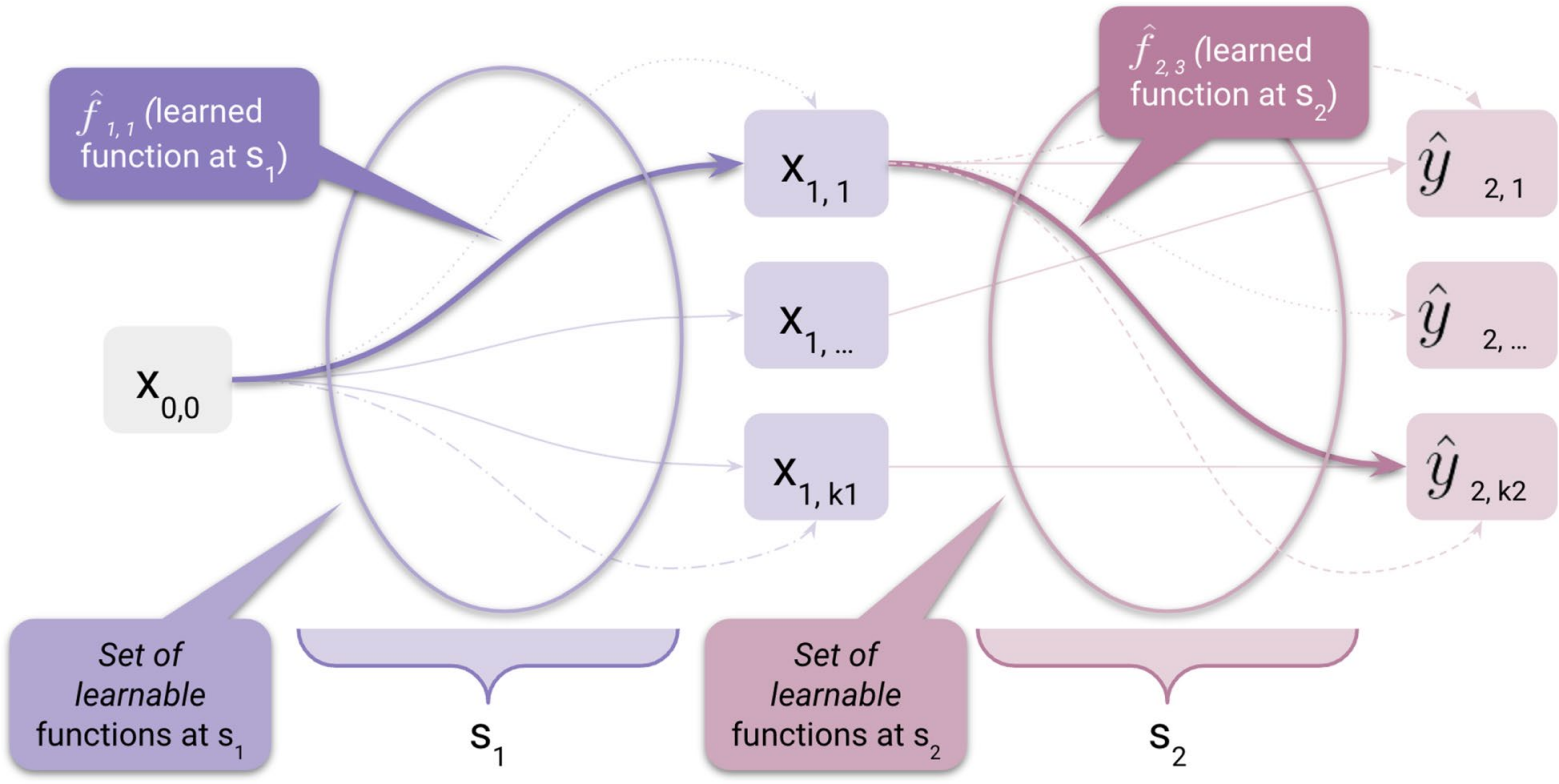
Example of transformation functions for two steps $s_i$

In this section, we formally characterize the inference process of a general ML model without any constraint on the task. Such a characterization will introduce the terminology that substantiates the main components of our proposed framework of explainability, whose details are provided in Defining explanations section. To this end, we define a ML model *M* as an arbitrarily complex function mapping a *model input* to a *model conclusion* through a sequential composition of *transformation steps*. The whole characterization is exemplified in Fig. 1.

### Definition 1

(Model Input) It is the set of features for a data point in the dataset, either derived from an observation or synthetically generated.

It is worth highlighting that the set of features could consist of any vectorial representation of a data point, e.g., pixel colors in an image and sub-word embeddings in a document representation. Thus, this definition is independent of the task to be solved or the data type.

### Definition 2

(Model Conclusion) It is the final output of the model, representing the outcome of the last link in the chain of transformations applied to the model input.

Sometimes the *model conclusion* is called *prediction*, or *inference*, or *forecast*. Since the aforementioned terms are somehow linked to the task to be solved, e.g., the term “forecast” should be used only for model conclusions that affect the future, we choose to use the broader term *model conclusion*. The model conclusion can thus be anything, depending on the task to be solved, e.g., a class probability (in a classification problem), a vector representing a word (in a next-word prediction task), or a simple value (in a regression problem).

### Definition 3

(Transformation Steps) Overall, the decision-making process of *M* can be represented as a chain of $N> 0$ transformations of the original model input that are causally related. This causal chain is enforced by the model design (e.g., the sequence of layers in a neural network’s architecture or the flow of a decision tree). We call each stage of this causal chain a “transformation step” and denote it with $s_i$, for $i\in [1, N]$. The transformation steps advance the computation from the model input to the model output through *transformation functions*.

### Definition 4

(Transformation Functions) Each transformation step $s_i$ relates to a set of $n_i$ “transformation functions” $f_{i,m_i}$, where $m_i\in [1,n_i]$ indicates one of the possible learnable functions at $s_i$. Note that, in general, the number of such functions would be infinite, but we discretize it, assuming we are working on a real scenario using some computational machine. The transformation functions are mappings from a feature set $x_{i-1,j}$ to a feature set $x_{i,z}$, with $j\in [1, k_{i-1}]$, $z\in [1, k_i]$ (i.e., the arrows enclosed in the ellipses in Fig. 1). The number $k_i$ denotes the cardinality of the set of all possible feature sets generated by all possible learnable transformation functions at step $s_i$. These transformation functions are generally opaque to the user in the context of the so-called black-box models. At every step in the chain of transformation steps, the model learns one of the possible transformation functions (i.e., the optimal function according to some learning scheme, highlighted with a solid line in Fig. 1). That is, the model learns the function $\hat{f}_{i,m_i}$ such that $\hat{f}=\hat{f}_{N,m_N} \circ \ldots \circ \hat{f}_{i,m_i} \circ \ldots \circ \hat{f}_{1,m_1}$ is the overall approximation of the true mapping from the model input to the model conclusion. According to the notation above, we denote the model input as $x_{0,0}$ (or simply *x*) and the model conclusion as $\hat{y}_{N,j}$, with $j\in [1, k_N]$ (or simply $\hat{y}$).

### Observations

We asserted that, at each transformation step $s_i$, the model picks one function $\hat{f}_{i, m_i}$ among $n_i$ such that $\hat{f}_{i, m_i}(x_{i-1,j})=x_{i,z}$. This raises issues that increase model opacity. At step $s_i$, the chosen function $\hat{f}_{i, m_i}$ can map different intermediate transformations $x_{i-1,j}$ of the feature set at the previous transformation step into the same transformation $x_{i,z}$ one step further in the chain. This means that the same outcome in the transformation chain, whether intermediate or conclusive, can be achieved through different rationales, and it may be difficult for a human user to understand which one the model has learned. This can result from a high cardinality of the set of transformation functions and a high complexity of the transformed feature set.

For example, pictures of zebras and salmon can be discriminated against based on their anatomy (i.e., zebras have stripes while salmon have gills) or the environment/habitat (i.e., zebras live in savannas and salmon in rivers). Consider a relatively complex model such as a Convolutional Neural Network (CNN), where a transformation step coincides with a layer within the network architecture. It is generally difficult to understand which kind of transformation $f_{i,m_i}$ this represents, much less if that is human-understandable. Thus, how do we understand which of the $n_i$ possible alternative mappings of $x_{i-1,j}$ led to $x_{i,z}$? This remains an open question with major implications for the discussion around faithfulness, which we will enlarge in the next section.

## Defining explanations

Recent work on ML interpretability has produced multiple definitions for the term “explanation”. According to Lipton, “explanation refers to numerous ways of exchanging information about a phenomenon, in this case, the functionality of a model or the rationale and criteria for a decision, to different stakeholders” cite Lipton2016spsee. Similarly, for Guidotti et al., “an explanation is an *interface* between humans and a decision-maker that is at the same time both an accurate proxy of the decision-maker and comprehensible to humans” cite Guidotti2018spsfw. Murdoch et al. add to how the explanation is delivered to the user, stating that “an explanation is some relevant knowledge extracted from a machine-learning model concerning relationships either contained in data or learned by the model. [...] They can be produced in visualizations, natural language, or mathematical equations, depending on the context and audience [18]. On a more general note, Mueller et al. state that “the property of being an explanation is not a property of the text, statements, narratives, diagrams, or other material forms. It is an interaction of (i) the offered explanation, (ii) the learner’s knowledge and beliefs, (iii) the context or situation and its immediate demands, and (iv) the learner’s goals or purposes in that context [34]. Finally, Miller tackles the challenge of defining explanations from a sociological perspective. The author highlights a wide range of explanations but focuses on those that provide an answer to a “why-question” [35].

**Fig. 2:**
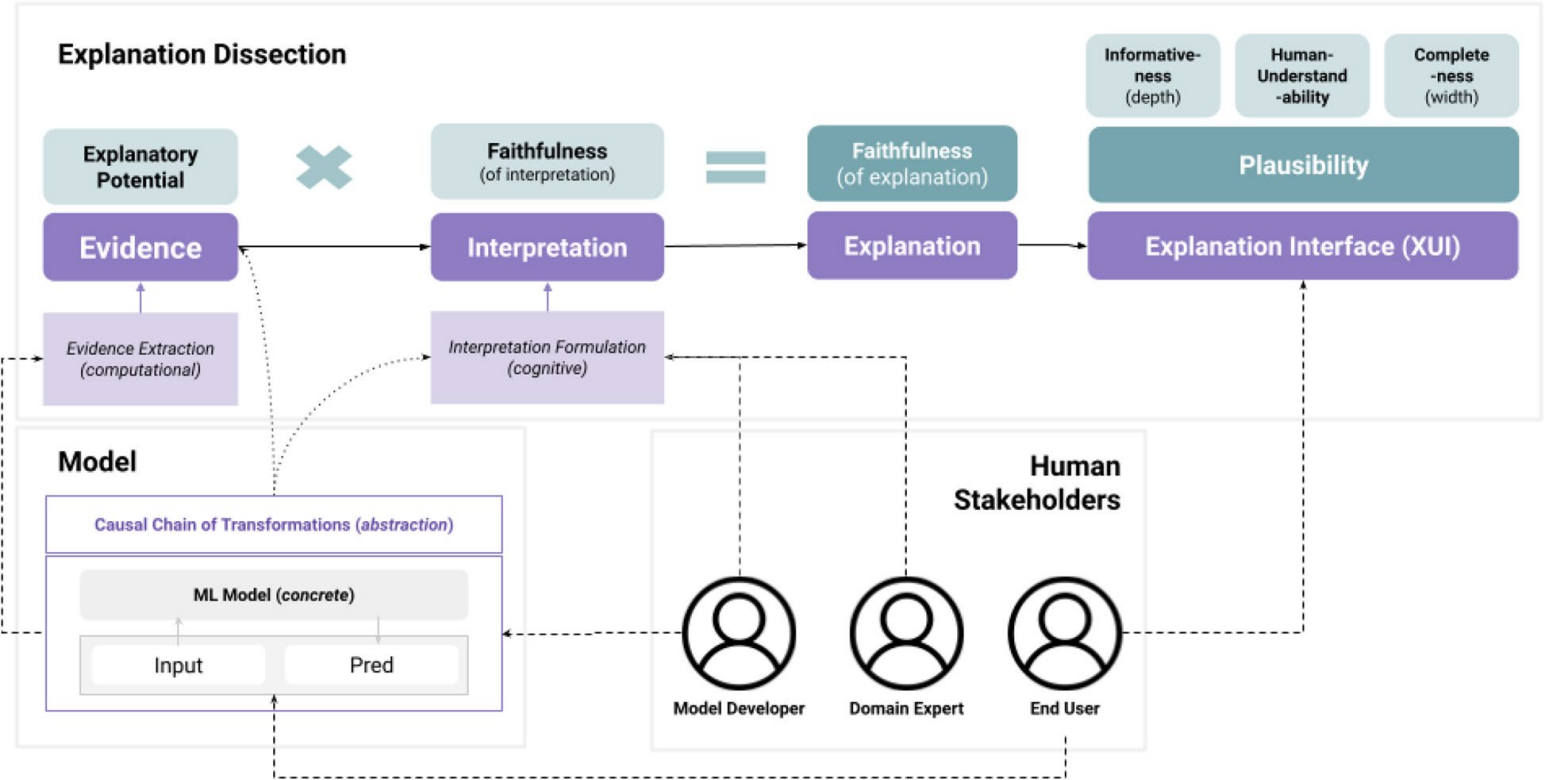
Overview of the theoretical framework of explainability

The definitions mentioned above offer a well-rounded perspective on what constitutes an explanation. However, they fall short in highlighting its atomic components and characterizing their relationships. We synthesize our proposed explanation definition by combining complementary aspects of existing definitions. The result is a concise definition that is easy to operationalize for supporting the analysis of multiple approaches to explainability. Our full proposed framework is reported in the scheme in Fig. 2. The next subsection will formally define its core components, i.e., evidence, interpretations, and explanations. The properties of these components, i.e., explanatory potential, faithfulness, human intuition alignment, and plausibility (with its sub-properties), are examined in Concerning faithfulness and plausibility section.

### Definitions of the components of the framework

#### Definition 5

(Explanation) Given a model *M* which takes an input *x* and returns a prediction $\hat{y}$, we define “explanation” as the output of an *interpretation function* applied to some *evidence*, providing the answer to a “why question” posed by the user.

#### Definition 6

(Evidence (*e*)) We define “evidence” (denoted by *e*) as whatever kind of objective information stemming from the model we wish to explain, and that can reveal insights into its inner workings and rationale for prediction (e.g., attention weights, model parameters, gradients, etc.).

#### Definition 7

(Evidence Extractor ($\xi$)) An “evidence extractor” (denoted by $\xi$) is a computational method fetching relevant information about *M*, *x*, $\hat{y}$, or a combination of the three. Then: $e = \xi (x, \hat{y}, M)$. Examples of evidence extractors are e.g., encoder plus attention layers, gradient back-propagation, and random tree approximation, with the corresponding extracted evidence being attention weights, gradient values, and random tree mimicking the original model. In the peculiar case of a white-box approach, that is, ML models designed to be “easily explainable” by the user (e.g., linear regression, fuzzy rule-based systems), the extraction of evidence is straightforward since all components of the model directly present a piece of semantic information in a human-comprehensible format.

#### Definition 8

(Explanatory Potential ($\epsilon (e)$)) We define “explanatory potential” (denoted by $\epsilon (e)$) of some evidence as the extent to which the evidence influences the causal chain of transformation steps of a model. Intuitively, the explanatory potential indicates “how much” of a model the selected type of evidence can explain. It can be computed either by counting the number of transformation steps impacted by the evidence (i.e., *breadth*) or by determining the extent to which each individual transformation step is impacted by the evidence (i.e., *depth*).

#### Definition 9

(Interpretation) An “interpretation” is a function *g* associating semantic meaning to some evidence and mapping its instances into explanations for a given prediction or the whole model. Then an explanation can be defined as either $E=g(e, x, \hat{y}, M)$, or $E=g(e, M)$, respectively.

#### Definition 10

(Explanation User Interface (XUI)) We define eXplanation User Interface (XUI) as the format in which explanations are presented to the end user. This could be, for example, in the form of text, plots, infographics, or other visual elements.

### Observations

#### Local vs. global interpretations

Following the existing literature, we relate “evidence” and “interpretation” to the concepts of *locality* and *globality*. Both evidence and interpretations can be either local or global in nature. Local evidence (e.g., attention weights, gradient, etc.) relates relevant model information to a particular model input *x* and corresponding prediction $\hat{y}$. Global evidence (e.g., full model parameters) is generally independent of specific inputs. It might explain the model’s higher-level functioning (providing deeper or wider info) or some of its sub-components. Similarly, interpretations can provide either a local or a global semantics of the evidence. A local interpretation of attention could be, e.g., “attention weights are descriptive of input components’ importance to model output”. On the other hand, a global interpretation of the same evidence may aggregate all the attention weights’ heatmaps for a whole dataset and highlight specific patterns. For example, in a dog *vs* cat classification problem, a global interpretation of attention may be represented by clusters of similar parts of the animal’s body (e.g., groups of ears, tails, etc.) highlighted by the attention activations.

#### Generating interpretations

Given some evidence involved in one or more steps $s_i$ of *M*, we “guess” how this evidence is involved in the opaque input-to-output transformations by formulating an interpretation *g* of some extent of the model’s decision-making process. At a low level, we generate a candidate hypothetical function *g* that encapsulates the approximations $f_{i,m_i}^* \tilde{=} \hat{f}_{i,m_i}$ of the behavior of certain functions learned by *M* at some steps $s_i$. On an abstract level, interpretations can be seen as hypotheses about the role of evidence in the explanation-generation process. Like a good experimental hypothesis, a good interpretation satisfies two core properties: (i) it is testable, and (ii) it clearly defines dependent and independent variables. Interpretations can be formulated using different forms of reasoning (*e.g.,* deductive, inductive, abductive, etc.). In particular, the survey on explanations and social sciences by Miller reports that people usually make assumptions (i.e., in our context, choose an interpretation) via social attribution of intent (to the evidence) [35]. Social attribution is concerned with how people attribute or explain the behavior of others, rather than the actual causes of that behavior. Social attribution is generally expressed through folk psychology, which involves attributing intentional behavior using everyday terms such as beliefs, desires, intentions, emotions, and personality traits. Such concepts may not truly be the cause of the described behavior, but are indeed those humans leverage to model and predict each other’s behaviors. This may lead to misalignment between a hypothesized interpretation of some evidence and its actual role in the inference process of the model. In other words, reasoning on evidence through folk psychology might generate interpretations that are *plausible* but not necessarily *faithful* to the inference process of the model (such terms will be further explored in Concerning faithfulness and plausibility section).

### Framework summary

As depicted in Fig. 2, our framework operates as follows: an *explanation* is derived from the *interpretation* of certain *evidence*. This evidence is produced by an *evidence extractor* that operates on the model in combination with its inputs and outputs. The expressiveness of the evidence can be characterized in terms of its *explanatory potential* (breadth and depth). This potential, along with the interpretation’s adherence to the model’s actual inference process (*faithfulness*), directly affects the overall faithfulness of the explanation. Finally, the *XUI* is responsible for conveying the explanation to the user.

## Concerning faithfulness and plausibility

### Faithfulness of interpretations and explanations

In the previous section, we observed that social attribution is a double-edged sword for the interpretation generation process, as it may propel plausibility without accounting for faithfulness. This issue was highlighted by Jacovi & Goldberg, who introduced a property of explanations called *aligned faithfulness* [24]. In the authors’ words, an explanation satisfies this property if “it is faithful and aligned to the social attribution of the intent behind the causal chain of decision-making processes”. Our proposed framework enables us to advance in characterizing this property. We note that the property of aligned faithfulness pertains only to interpretations, not evidence. The latter has no inherent meaning. Its semantics are defined by some interpretation that may or may not involve social attribution of intent to the causal chain of inference processes.

#### Definition 11

(Faithfulness (of interpretation)) Given an interpretation function *g*, describing some transformation steps $s_i$ within a model *M*’s inference process, we want to be able to prove that *g* is faithful (at least to some extent) to the actual transformations made by *M* to an input *x* to get a prediction $\hat{y}$. Namely, we define the property of *faithfulness of an interpretation* $\phi _i(g, e)$ as “the extent to which an interpretation *g* accurately describes the behavior of some transformation functions $f_{i,m_i}$ that some model learned to map an output $x_{i-1, j}$ at $s_{i-1}$ into $x_{i,z}$ at $s_i$ making use of some instance evidence *e*”.

#### Definition 12

(Faithfulness (of explanation)) Given some evidence *e* and its interpretation function *g*, we say that a related explanation is faithful to some transformation steps if the following conditions hold: (i) the evidence *e* has explanatory potential $\epsilon _i>0$, and (ii) the interpretation *g* has faithfulness $\phi _i>0$. Then, we can define the *faithfulness of an explanation* ($\Phi$) as a function of the faithfulness of the interpretation of each step involved and the related explanatory potential.

For example, we could define $\Phi = \sum \nolimits _i \epsilon _i \phi _i ~\forall i \in I \subseteq [1, N]$ where *I* is the set of indices of transformation steps $s_i$ that involved the evidence *e*. Thus, the faithfulness of an explanation is the sum of the faithfulness scores of its components, i.e., the faithfulness of the interpretations of the evidence involved in generating the explanation. Additionally, the related explanatory power weights the faithfulness of each interpretation, following the intuition that evidence with a higher $\epsilon$ value should have a greater impact on the overall faithfulness score of the interpretation. We can have various measures of faithfulness associated with different explanation types, in the same way that we have different metrics to evaluate the ability of an ML model to complete a task. Thus, $\phi _i$ is implicitly bounded.

When designing a faithful explanatory method, we can opt for two approaches. We can achieve faithfulness “structurally” by enforcing this property on pre-selected interpretations in model design (e.g., imposing constraints on transformation steps that limit the range of learnable functions). This direction has been recently explored by Jain & Wallace [36] and Jacovi & Goldberg [24]. An alternative, naive strategy is trial and error: formulating interpretations and assessing their faithfulness via formal proofs or requirements-based testing using proxy tasks. While formal proofs are still missing in the current literature, several tests for faithfulness have been recently proposed [12–14, 37–39].

### Plausibility of the explanation user interface

Explanations are intended for delivery to specific target users, and that is when XUIs come into play. We argue that an XUI is characterized by three main properties: (i) human understandability, (ii) informativeness, and (iii) completeness. The *human-understandability* is the degree to which users can understand the answer to their “why” question via the XUI. This property depends on user cognition, bias, expertise, goals, etc., and is influenced by the complexity of the selected interpretation function. The *informativeness* (i.e., depth) of an explanation is a measure of the effectiveness of an XUI in answering the why question posed by the user. That is the depth of information for some $s_i$ of great interest in the XUI. The *completeness* (i.e., width) of an explanation is the extent to which an XUI describes the overall model’s workings and the degree to which it allows for anticipating predictions. That is the width in terms of the number of $s_i$ the XAI spans. Note that informativeness and completeness are bound by the explanatory potential of the evidence (e.g., attention weights do not explain the entire model, just some transformation steps; in contrast, the complete set of model parameters does). The combined value of the three properties mentioned above of XUIs drives the plausibility of an explanation.

#### Definition 13

(Plausibility (of explanation)) We define *plausibility* as the degree to which an explanation is aligned with the user’s understanding of the model’s partial or overall inner workings.

**Fig. 3:**
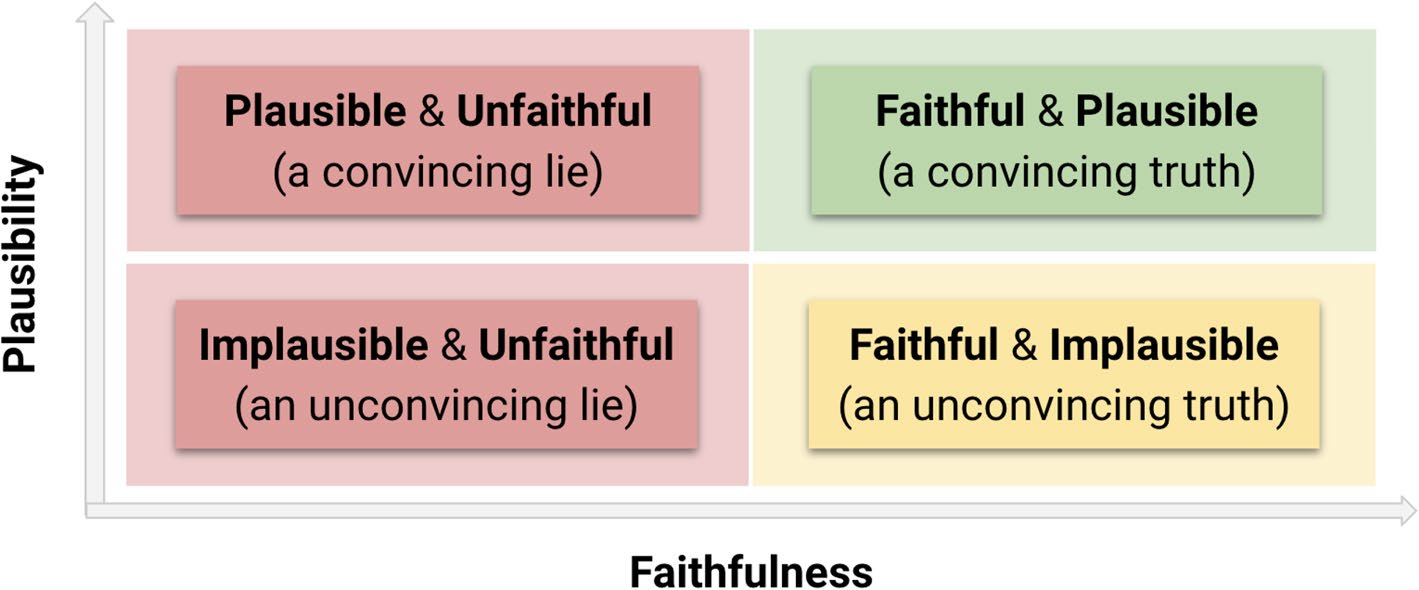
Overview of the outcome on the user of the interaction between faithfulness and plausibility

Plausibility is a user-dependent property, and as such, it is subject to the user’s knowledge, bias, etc. Unlike faithfulness, the plausibility of explanations can be assessed via user studies. Note that a plausible explanation is not necessarily faithful, just like a faithful explanation is not necessarily plausible. It is desirable for both properties to be satisfied in the design of some explanation. Interestingly, an unfaithful but plausible explanation may deceive a user into believing that a model behaves according to a rationale when, in fact, it does not. This raises ethical concerns around the possibility that poorly designed explanations could spread inaccurate or false knowledge among the end-users. Figure 3 provides a simplified problem overview.

### Connecting the dots: abductive reasoning

Abductive reasoning, a pivotal concept in XAI, is crucial in generating and interpreting explanations for ML models [40]. This form of reasoning enables the inference of a condition to explain an observed consequence. It is deeply entrenched in the philosophy of science and particularly resonates with the works of Charles Sanders Peirce. Peirce’s perspective on abduction as a logical inference that generates new hypotheses is foundational in this context [41]. The application of abductive reasoning spans various domains. In medical diagnostic AI, for instance, it assists in deducing potential health conditions from symptomatic data and patient history, enhancing the trustworthiness and dependability of AI recommendations in healthcare [42]. In AI, abductive reasoning primarily elucidates the ‘why’ behind AI decisions and predictions. It involves constructing *plausible* hypotheses from observed data, followed by iterative refinement through testing and validation [43]. Or, in our words, it involves building interpretations of the evidence aligned with human intuition. Explainable methods usually fall short at this point, when human intuition surpasses the importance of faithfulness in the interpretation. We advocate for a focus on faithfulness, ensuring the interpretation accurately represents the model’s decision-making. *Alignment* with human intuition is a nice-to-have for the interpretation but a necessity for the final explanation, which is why it must be considered when designing the XUI. A significant challenge in XAI is making the abductive reasoning processes *understandable* to users. Designing XAI interfaces that effectively communicate the AI’s hypotheses and reasoning enhances user understanding and trust, especially in high-stakes domains like healthcare [44].

Finally, abductive reasoning in AI is dynamic, evolving with new data and insights. This necessitates AI systems that can adapt and refine their explanations over time, underlining the importance of continuous learning and adaptation in AI technology. Abductive reasoning plays a multi-dimensional and vital role in ML models, extending beyond hypothesis generation to provide clear and understandable explanations, thereby bolstering the usability and reliability of AI systems.

## Case studies: framing common explainability strategies

In this section, we apply the theoretical framework to existing *explainability* methods. Additionally, we showcase the application of this framework to two “easily explainable” methods, namely linear regression and fuzzy models. Our objective is to provide concrete examples of how various XAI methods nicely fit into the proposed framework’s components. We also want to emphasize the usefulness of a common set of terms to indicate different concepts frequently encountered in XAI discourse. Despite our examples being limited to some of the most influential methods, we believe these examples will provide sufficient guidance for future research that is willing to apply this framework.

### Attention

The introduction of attention mechanisms has been one of the most notable breakthroughs in DL research in recent years. Originally proposed for empowering neural machine translation tasks [11], it is currently employed in many state-of-the-art approaches for numerous cognitive tasks, especially as the core of the ubiquitous Transformer architecture [25]. The chain of transformations in the simplest neural model making use of self-attention (one of the most common nuances of this strategy) is a three-step causal process: (i) encoding, (ii) weight encodings by attention scores, and (iii) decoding into model output. In particular, during the *encoding* phase, the input features are represented into *t* related tokens. Then, from the tokens, the three main components of the weighting procedure are created: the queries (*Q*), the keys (*K*), and the values (*V*). The term *self* in self-attention means that *Q*, *K*, and *V* are actually the same, and we denote them with $\bar{X}$. Usually, the vectors in $\bar{X}$ are then linearly projected with three different linear applications, one for each component, that we omit for the sake of clarity. Then, in the second phase, a weighted sum between the values in $\bar{X}$ is computed, where each weight is derived by the similarity between the vector in the queries and the vector in the keys. The similarity values are generally called *attention weights* since they represent the “attention” given to each input vector. Then, in the third phase, the newly generated vectors are decoded into new tokens. Then we can define the function learned by the model as the composition $\hat{f} = f_{3, m_3} \circ f_{2, m_2} \circ f_{1, m_1}$, where each $f_i$ for $i\in [1,3]$ corresponds to the respective transformation function in the causal chain.

**Evidence.** For an input *x* split into *t* sequentially related tokens, let $f_{1, m_1}$ be an encoder function such that $f_{1, m_1}(x)=\bar{X}$ is the vector of the encoded model input tokens. Then, $f_{2, m_2}(\bar{X})=\sum \nolimits _{j=1}^t \alpha _j \bar{x}_j$, for all model input tokens $\bar{x}_j \in \bar{X}$, is the combination (that we assume to be linear for demonstration purposes, aware of the fact that many nuances of attention exist) of the encodings weighted by their corresponding attention scores. Then $e_{att} = \{\alpha _j\}_1^t$.

1 $$\begin{aligned} e_{att} = \{\alpha _j ~| ~f_{2, m_2}(\bar{X}) = \sum \limits _{j=1}^t \alpha _j \bar{x}_j\} \end{aligned}$$

The evidence $e_{att}$ related to a model input is the set of weights $\alpha _j$ produced by the attention layer. The explanatory potential $\epsilon (e_{att})$ is the ratio between the number of parameters involved in the analyzed attention layer and the total number of parameters of the model.

#### Interpretation

The interpretation of the evidence is a function $g_{att}(e(x,\hat{y}))$ that describes function $f_{3, m_3}$, i.e., how the weighted encodings are decoded into the model conclusion.

#### Faithfulness

Note that we do not know the faithful interpretation function, so we hypothesize its behavior by formulating a candidate interpretation, a process usually guided by the researcher’s intuition. In the case of attention, an interpretation generally shared among researchers is that “the value of each attention weight describes the importance of the corresponding token in the original input to the model output”. Unfortunately, although plausible, research in this field disproved such an interpretation of attention weights [12, 14, 45], leaving the role of attention for explainability (if any) still unclear.

### Grad-CAM

A popular explanation called Grad-CAM [26] presents a method to explain a prediction made by an image classifier using the information encompassed in the back-propagated gradient of a prediction. Grad-CAM uses the information about the gradient computed at the last convolutional layer of a CNN, given a certain input *x*, to assign a feature importance score for each input feature. In brief, Grad-CAM computes a so-called “class-discriminative localization map” for each class in the classification problem. That is, for each class of the classification problem (e.g., cat, dog, etc.), Grad-CAM creates a matrix with a height and width equal to the input matrix, and for each pixel (or input feature), it associates a value that is related to the positive or negative influence of the pixel to the final prediction. Those values are computed starting from the gradient of the score for a class with respect to the feature activation map of a convolutional layer.

#### Evidence

The Grad-CAM evidence-extraction $\xi _{grad}$ method consisted of using the feature activation map of a convolutional layer from a given input *x* to compute the neurons’ importance weights $\alpha _{i}$. The explanatory potential $\epsilon (e_{grad})$ is related, as for the attention mechanism, to the number of parameters analyzed with respect to the total number of parameters of the method.

#### Interpretation

Grad-CAM claims that the computed neuron’s weights $\alpha _i$ correspond to the part of the input features that influence the final prediction the most.

#### Faithfulness

The authors measure the faithfulness of the model using image occlusion. They modified certain aspects of the model’s input and measured the correlation with the resulting difference in the final output. With this faithfulness metric, a high correlation means a high faithfulness in the explanation.

### SHAP

Lundberg & Lee in 2017 proposed SHAP [10], a method to assign an importance value to each feature used by an opaque model *M* to explain a single prediction $\hat{y}$. SHAP has been presented as a generalization of other well-known explanation methods, such as Local Interpretable Model-agnostic Explanations (LIME) [9], DeepLIFT [46], Layer-wise relevance propagation [47], and classic Shapley value estimation [10]. The SHAP values are defined as:

2 $$\begin{aligned} h\left( z^{\prime }\right) =\beta _0+\sum \limits _{i=1}^M \beta _i z_i^{\prime } \end{aligned}$$

where $z_i^{\prime } \in \{0,1\}^M$ is a simplified version of the input *x*, *M* is the number of features used in the explanation, and $\beta _i \in \mathbb {R}$ is a coefficient that represents the effect that the $i-th$ feature has on the output.

#### Evidence

The only evidence $e_{shape} = \xi _{shap}(M, x)$ used by (the original version of) SHAP is the set of predictions made by the classifier in a neighborhood of *x*. To compute the explanatory potential $\epsilon \left( {e_{shap}}\right)$, we can use the ratio of predictions employed to compute the SHAP values w.r.t the total number of possible samples in the countable (and possibly infinite) neighborhood of *x*. Thus, the greater the number of predictions we have, the higher the exploratory potential of the method.

#### Interpretation

The interpretation $g_{shap}$ of the evidence proposed by SHAP is that, given $e_{shap}$, we can locally reproduce the behavior of a complex unknown model with a simple additive model $h\left( \cdot \right)$, and analyzing $h\left( \cdot \right)$ we can get a local explanation $E_{shap}$ of the behavior of the initial model. The proposed interpretation of the evidence results from the optimization problem in Eq. (2).

#### Faithfulness

Even though the authors do not present a measure of the faithfulness of the explanation directly, they provide three desirable properties that are *i)* local accuracy, *ii)* missingness, *iii)* consistency. The authors showed that their method is the only one that satisfies all these properties, assessing a requirements-based form of faithfulness as described in Concerning faithfulness and plausibility section.

### Linear regression models

Linear regression models are not an explanation method but are normally considered *intrinsically interpretable*. Following our proposed framework, we argue that defining them, along with other models, as *intrinsically interpretable* is inaccurate and often misleading. The definition of what is simple to interpret by humans is not well-defined, and we can enumerate various examples of models that are easy to interpret for practitioners but are almost black-boxed for non-expert users.

A linear regressor $\hat{f}_{lin}(\cdot )$ is typically formulated as:

3 $$\begin{aligned} \hat{f}_{lin}(x)=\beta _0+\sum \limits _{i=1}^N \beta _i x_i^{\prime } \end{aligned}$$

where $\beta _i$ are the weights of the learned features, and *N* is the feature space dimension.

#### Evidence

The implicit assumption, claiming that a linear model is intrinsically interpretable, is that the weights ${\beta _i, 1 \le i \le M}$ are a good explanation for the model. Thus $e_{lin} = \{\beta _i\}_1^N$. We have the maximum explanatory potential $\epsilon(e_{lin})$ with a linear model because we can fully describe the model with $e_{lin}$.

#### Interpretation

Assuming a normalization of the features, we can say that the higher the value of $\beta _i$, the higher the contribution of the feature $x_i$ to the model prediction.

#### Faithfulness

There are no doubts about the faithfulness of the interpretation of the predictions given the normalization assumption, and in fact, a linear model is normally considered an intrinsically interpretable method. However, a real scenario does not guarantee its plausibility to a non-expert user.

### Fuzzy models

Fuzzy models, especially in the form of Fuzzy Rule-Based Systems (FRBS), represent effective tools for modeling complex systems using a human-comprehensible linguistic approach. Thanks to these characteristics, they are generally considered white or gray boxes and often considered good options for interpretable AI [48]. Although a detailed description of fuzzy modeling goes beyond the scope of this paper, it is important to specify that FRBSs perform their inference (i.e., calculate a conclusion) by exploiting a knowledge base composed of linguistic terms and rules. Thanks to this linguistic approach and the fact that fuzzy set theory can naturally embed uncertainty and vague concepts, FRBS is generally considered *intrinsically interpretable* models. A fuzzy rule is usually expressed as a sentence in the form:

4 $$\begin{aligned} \texttt {IF < antecedent > THEN < consequent >} \end{aligned}$$

where antecedent is a logic formula created by concatenating clauses like X IS a with some logical operators, where T is a linguistic variable (associated with one input feature) and *a* is a linguistic term. Thanks to this representation, the antecedent of each rule provides an intuitive and human-understandable characterization of a specific class/group.

The form of consequent varies according to the type of model and fuzzy reasoner used. Still, it can be seen as a function that calculates the model conclusion such that the more a sample satisfies the antecedent, the higher the weight of this rule in the final calculation of the model conclusion. Note that, due to the fuzziness of the model, all rules can be applied simultaneously, although with different weights.

#### Evidence

The rules are good evidence for a large part of the model: they characterize the feature space using a self-explanatory formalism that human operators can read and validate. The fuzzy terms are implemented using fuzzy sets and corresponding membership functions, which are typically parametric curves, often in the form of triangles, trapezoids, sigmoids, or Gaussian functions.

#### Interpretation

The fuzzy sets used to create the fuzzy terms and evaluate the satisfaction of the antecedents have self-explanatory interpretations: they define how much a value belongs to a given set employing membership functions. The fuzzy rules are also self-explanatory. The only part that requires a proper interpretation is the output calculation function. In the case of Sugeno reasoning, such functions can be viewed as linear regression models; hence, all considerations discussed in Linear regression models section remain valid in the context of fuzzy models.

#### Faithfulness

Similarly to the case of linear regression models, there are no doubts about the faithfulness of the interpretation of the predictions given a normalization step. However, in the case of special transformations (e.g., log-transformation), some of the intrinsic interpretability might be lost in favor of better fitting to training data [48]. Since it is often the case that features in biomedicine (see, e.g., clinical parameters) follow a log-normal distribution, such transformations are very frequent and delicate.

## The role of the user

### Evidence, interpretations, and explanations: who owns what?

The concept of ownership in explanations is multifaceted and central to understanding explainable ML. In the so-called “intrinsically explainable models” (or, as we discussed, “more easily explainable models”), the system naturally generates a component of the explanations as part of its processing. This is the evidence, factual data that emerges directly from the model’s decision-making process. The ownership of this objective information belongs to the model, as it is a product or by-product of the model. However, identifying tangible data from the model as evidence is part of the human process of designing the explanation. While the model generates the initial evidence, the final understanding and contextual arrangement of this data are undertaken by human users. This implies dual ownership. The system ‘owns’ the initial explanatory data. It provides an opportunity for human stakeholders to identify this data as the evidence on which an explanation is built. Identifying the evidence is the human (owned) process of relating some intuitive explanatory potential to information stemming from the model.

Similarly, the ultimate interpretation and the meaning derived from this evidence are ‘owned’ by the human users. In this context, ownership is also closely tied to responsibility. The human users are responsible for interpreting the evidence within the context of their domain knowledge and the specific situation at hand. To tap into the philosophy of linguistics and borrow from the basics of the semiotic triangle [49], we might call the data itself the *signifier*, the evidence the *referent*, and the interpretation the *signified*. The semiotic triangle illustrates the relationship between a concept (the “signified”), the physical form or symbol that represents it (the “signifier”), and the real-world object or idea to which it refers (the “referent”), highlighting how meaning is constructed in human language and thought. For a real-world example in the context of ML explainability, the semiotic triangle can be applied to the concept of a “feature importance” score in a decision tree model. In this case, the signified is the concept or idea of “feature importance” (an interpretation), which represents the relative importance or contribution of an input feature to the prediction made by the model; the signifier is the numerical score or graphical representation, e.g., a bar chart, (the data) used to indicate the importance of each feature in the model’s decision-making process; the referent is the actual data attribute or input variable to which the feature importance score is applied (the evidence), influencing the model’s predictions in the real world.

This shared ownership representation raises critical questions about the design of AI systems and the type of explanations that need to be generated. It necessitates a design approach that considers not only the technical capability of AI systems to provide explanatory data but also the ability of human users to identify this data as such and interpret it effectively.

Ownership becomes even more nuanced when considering models that are harder to explain, the so-called “black-box” models. These models, such as deep neural networks, do not naturally provide easy access to explanatory information for their decisions. As a result, external methods, known as post-hoc explainability techniques, are employed to interpret the model’s behavior. Here, the ownership of explanations is even more distributed. The AI system still ’owns’ the decision-making process but does not inherently own all the explanatory data, as these are not completely a product or by-product of the model. Instead, the explanations are generated through additional tools and methods that produce explanatory information from initial data stemming from the model and are then interpreted (e.g., SHAP). These tools aim to provide a proxy context for examining and explaining the model’s decision-making process, but they introduce an additional layer of abstraction and potential bias. Developers of these tools are responsible for ensuring that the post-hoc methods accurately represent the model’s decisions, while users must critically assess the validity and relevance of these explanations. In this case, the ownership of the explanation is shared among the AI model (raw evidence), the explainability tool (processed evidence), and the human interpreters.

### Explainers, explainings, and explainees

In the academic exploration of ML explainability, a progressive understanding of the roles of the explainer and the explainee has been developed, emphasizing the dynamic and interactive nature of this relationship. The discourse initially focused on the need for explainers (AI systems) to provide comprehensible and relevant explanations to explainees (human users), especially in high-stakes fields such as healthcare. Early research underscored the responsibility of explainers to bridge the gap between complex ML models and the practical needs of domain experts, thereby enhancing the explainee’s ability to make informed decisions. The narrative has evolved with the introduction of frameworks that shifted towards interactive explainability. For instance, Rovolis et al. recently explored the impacts of participatory design on data-driven decision-making in organizations, highlighting the integration of participatory activities into decision-making processes to achieve better outcomes [50]. This approach blurred the traditional boundaries between explainer and explainee, suggesting a more collaborative and iterative understanding of ML models. The concept of personalized explainability emerged, underscoring the importance of tailoring explanations to the individual needs and contexts of explainees. This perspective recognizes the diversity in the user base of automated systems and the necessity for explainers to adapt their outputs accordingly. For example, Gould et al. investigated patients’ views on AI for risk prediction in shared decision-making for knee replacement surgery, emphasizing the need for AI tools to provide personalized information that empowers patients and supports a partnership between clinicians and patients [51].

Further contributions focused on evaluating explainers in educational contexts and designing user studies to assess the effectiveness of explanations. These studies emphasized the explainer’s role in being comprehensible to non-expert users and the active role of the explainee in editing and understanding ML models. The conventional wisdom of ranking ML algorithms based on their explainability was challenged, advocating for a more nuanced, user-centered approach. This shift recognized the varied needs and contexts of explainees, suggesting that the effectiveness of an explainer should be assessed based on its relevance and utility to the specific user. Most recently, the integration of explainable AI methods into the learning loop and the potential of explanations for eliciting user control and feedback were discussed. These studies highlighted the evolving nature of the explainer-explainee relationship, where explanations are a one-way communication tool and a means for enhancing model performance and user understanding. In conclusion, the scholarly works in this field collectively advance the understanding of the roles of explainer and explainee in ML explainability. They reflect a shift from a unidirectional flow of information to a more interactive, user-centric model, recognizing the importance of context, personalization, and active user engagement in explainable AI.

**Fig. 4:**
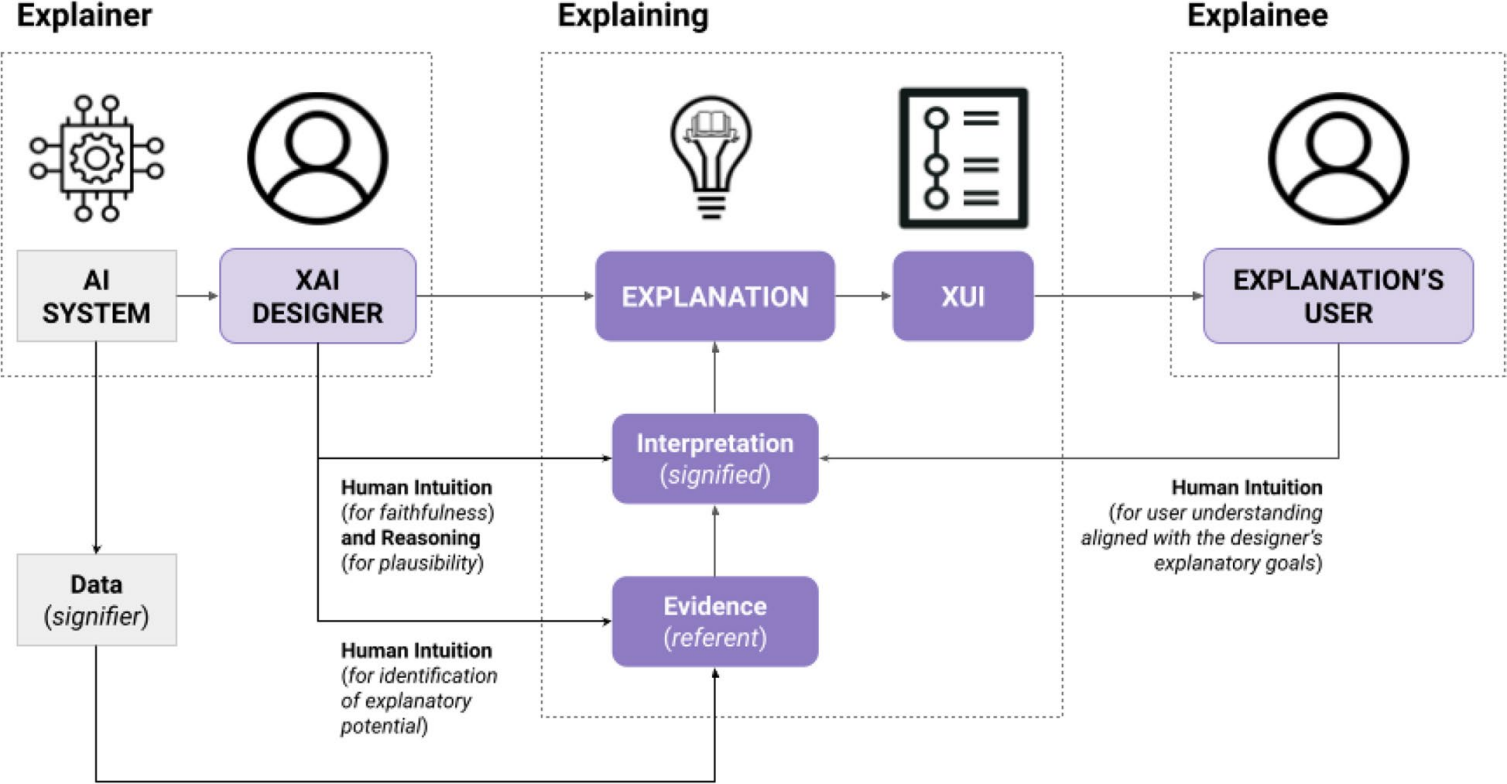
Our proposed relationships among the explainer, the explaining, and the explained in ML explainability

Regarding our contribution, based on our novel framework, we propose a shift in perspective. Our proposal is synthesized in Fig. 4. The explainee remains the user of the human explanation. On the other hand, we argue that the explainer is not only created by the AI system but also by an admixture of human knowledge, design, and raw data. Unlike previous work, we focus on the role of the explainer within a human-machine pair, where the human plays the active role. The human is the designer of explainability for a given ML model. This challenge may be more or less difficult depending on the circumstances (e.g., the model type to explain, the degree of designer intervention in the model architecture, etc.), and we will discuss this in-depth in the next section.

Before that, we need to define a novel third component to enrich the discussion around the core roles of the explainability pipeline. We refer to this component as the “explaining”, which has the concrete role of explaining. The “explaining” embeds the information that the explainee needs to understand in order to grasp the model’s decision-making process. To our knowledge, this role is usually understated and remains a simple by-product of human design. We argue that this component deserves space on its own as it is crucial to the discussion around the explainer-to-explainee knowledge communication. The “explaining” comprises the explanation, made out of its atomic components previously discussed in this paper (evidence and interpretation), and the final overlay of the explanation user interface that relates directly to the explanation target user. The human role is key to defining “the explaining”. First, the XAI designer must use human intuition to identify the explanatory potential in the evidence. This process turns raw data from the AI system into actionable data for producing an explanation. Second, intuition and human reasoning are needed to formulate an interpretation. Third, the explanation user must resonate with the interpretation, meaning that he must be able to use human intuition to understand (at a certain, not necessarily full, degree) the rationale for an explanation [52]. This goes back to the debate concerning faithfulness and plausibility we discussed in Concerning faithfulness and plausibility section.

The dynamics between the explainer and the explainee are crucial in AI explainability. The explaining mediates these. In the case of more explainable models, the AI system, as the explainer, provides easy-access information for explaining its decision-making. However, the role of the explainer is not just to provide raw data or evidence; it also includes analyzing its meaning and presenting this information in a manner that is accessible and comprehensible to the human user. This involves considering the user’s background, expertise, and the context in which the AI system is used. Therefore, the explainer’s role is not passive but actively involves tailoring the explanation to suit human requirements. It becomes clear that it is of the utmost importance for AI systems and human designers to collaborate across each stage of the AI product delivery process, from blueprints to output, to achieve explainability. The explainee, typically the human user, engages with the explanation provided by the AI system. The explainer’s role involves interpreting, understanding, and contextualizing the explanation within their domain of expertise. This is not a straightforward process, as it requires the human user to apply their knowledge, experience, and judgment to make sense of the information provided by the AI system. The explainee’s role is critical in ensuring the explaining is understood, actionable, and relevant to the decision-making process.

Sometimes, the role of the explainer is bifurcated between the AI system and the explainability tools. The AI system executes the decision-making process, while the tools act as translators, making these decisions understandable to human users. This two-step explanation process complicates the explainer’s role, as it introduces potential discrepancies between the model’s actual decision-making process and how the tools represent it. The explainee’s role also becomes more challenging in this context. Human users must understand the explanations provided by these tools, as well as their limitations and potential biases. This requires higher critical thinking and awareness of the explainability methods used. Furthermore, some models often need iterative feedback between the explainee and the explanation system. This iterative process helps refine and tailor the explanations to the user’s needs and understanding. It underscores the dynamic nature of the explainability process in AI systems, where the explanations are not straightforward or inherently clear.

In summary, the roles of the explainer and explainee are interdependent and collaborative. While the AI system generates the initial data, the human user plays a vital role in diagnosing and applying this information. This collaboration is essential for the effective use and trust of AI systems, particularly in complex domains where decisions have significant implications. The separation between the decision-making process and explanation generation introduces multiple layers of responsibility. It requires a collaborative and iterative approach between the AI system, the explainability tools, and the human users. Understanding these dynamics is crucial for effectively implementing and trusting AI systems in complex, high-stakes domains.

## On the implications for biomedicine

### Understanding evidence and interpretation in biomedical decision-making

The interplay between evidence and interpretation is fundamental in utilizing and understanding ML models in biomedical decision-making. ’Evidence’ in this context refers to the diverse and complex data types processed by ML models, ranging from genomic sequences and clinical metrics to imaging data and unstructured clinical notes. These data types are often high-dimensional and intricate, necessitating advanced ML models for meaningful pattern extraction. ’Interpreting’, conversely, is the process of contextualizing this evidence. It involves translating the model’s output into actionable insights by integrating it with clinical knowledge, patient history, and current medical research. This translation is not merely a technical process but a critical bridge between AI and clinical expertise [53]. One of the primary challenges in this domain is ensuring that the evidence provided by ML models is detectable to clinicians. Simplifying complex data without losing critical information is vital to making these models useful in practical settings. Techniques such as visualization tools and feature importance metrics significantly transform intricate data patterns into more digestible formats, highlighting the aspects of data that most significantly influence the model’s predictions [54]. The field is evolving towards more sophisticated methods for presenting evidence and facilitating its interpretation. This includes the development of algorithms that can automatically highlight clinically relevant features in complex datasets and the creation of interfaces that allow clinicians to interact more intuitively with the model’s outputs [55].

Balancing evidence and interpretation is crucial for successfully applying ML in biomedicine. By enhancing the clarity and relevance of the evidence presented by these models and improving the tools and techniques for its interpretation, we can bridge the gap between AI capabilities and clinical needs. This advancement leads to more effective and trustworthy biomedical decision-making, aligning technological innovation with the nuances of clinical practice [53].

### Elevating trust and precision in biomedical decisions through faithfulness

Integrating sophisticated ML models in biomedicine has been transformative, offering unprecedented capabilities in diagnosing and predicting medical conditions [53]. However, the complexity of these black-box models necessitates explanations that are not only accurate but also deeply insightful and faithful. Such faithfulness in explanations is crucial for ensuring transparency and justifiability in medical decision-making. ML models revolutionize diagnosis and prognosis in high-stakes fields like oncology, cardiology, and neurology. Decisions derived from these models, particularly in cancer treatment, require a comprehensive analysis of patient-specific data, including genetic markers and tumor characteristics [56]. The depth of understanding provided by faithful explanations enables oncologists to validate AI recommendations, which is essential for patient trust and improved outcomes [55].

Furthermore, the importance of faithful explanations extends to error identification and model refinement. In biomedicine, where errors can have life-altering consequences, understanding the decision-making process of ML models is invaluable. This understanding is crucial for identifying sources of error, such as biases in training data or overfitting issues [45]. Regulatory bodies, such as the FDA, also focus on the explainability and transparency of AI systems in medical devices and software, making faithful explanations a key component in meeting regulatory standards [57]. In clinical environments, where decision-making is often collaborative, faithful explanations of ML model decisions provide clarity to all team members, fostering enhanced discussions and more informed patient care strategies [58]. This clarity is beneficial for current clinical practice and holds significant educational value. Understanding the rationale behind AI-driven decisions is a powerful learning tool for clinicians and medical students, offering insights into novel diagnostic or therapeutic approaches [59]. Patient involvement in healthcare decisions is increasingly emphasized, and the role of faithful explanations in enhancing patient trust and engagement is significant. When patients understand how an AI system has contributed to their diagnosis or treatment plan, their trust in the care regimen improves [60]. The journey towards a more transparent, reliable, and patient-centered healthcare system is closely tied to the faithfulness of ML model explanations. Future developments in AI for biomedicine should focus on improving predictive accuracy and enhancing the faithfulness of their explanations, bridging the gap between advanced technology and practical clinical application [61].

### Bridging the gap between expertise and explainability through plausibility

The concept of plausibility in model explanations is pivotal in rendering complex ML models accessible and comprehensible to a diverse group of stakeholders in biomedicine, ranging from clinicians and researchers to patients and policymakers. Plausibility plays a crucial role in translating the intricate technical details of a model’s workings into actionable clinical insights, making it an essential component in scenarios such as patient diagnosis or treatment planning [62]. In the context of patient-centered care models, where patient engagement and informed decision-making are paramount, the plausibility of explanations is particularly significant. Simplified yet accurate explanations help patients understand their medical conditions and the rationale behind specific treatments or recommendations, thus enhancing their engagement in their care [63]. This plausibility aspect is crucial for patient communication and facilitating interdisciplinary collaboration in modern healthcare. Teams of professionals with varying degrees of technical expertise rely on plausible explanations to ensure that AI-driven insights are accessible and meaningful to all members, enabling collaborative and informed decision-making [64].

Moreover, plausible explanations address ethical and legal considerations by ensuring transparency in decision-making processes. As AI in healthcare faces increasing scrutiny and regulation, providing clear and understandable explanations of how AI models arrive at their conclusions is a legal and ethical imperative [65]. However, creating plausible explanations presents challenges. The risk of oversimplification, where key details might be lost to make the explanation more accessible, is a significant concern. Additionally, the need to cater to different stakeholders, each requiring different levels and types of explanation, adds complexity to designing universally plausible explanation systems [66]. Future directions in enhancing plausibility in explanations should focus on developing adaptive systems that can tailor explanations to the needs of different users. Establishing guidelines to balance technical accuracy with user accessibility in explanations is crucial in the biomedical domain [67]. This approach will bridge the gap between technical expertise and interpretability, fostering trust, collaboration, and ethical compliance in the use of AI in biomedicine.

### Navigating the faithfulness-plausibility trade-off

The interplay between faithfulness and plausibility in ML explanations is a nuanced and critical aspect, particularly in biomedicine. Faithfulness ensures that explanations accurately represent the model’s decision-making process, which is essential for maintaining scientific rigor and ensuring clinical safety. Conversely, plausibility ensures that these explanations are interpretable and meaningful to end-users who may not have technical expertise [67]. This balance is particularly challenging in biomedical data, which often involves intricate and multifaceted information. For example, a faithful explanation for a genomic analysis model must detail intricate pathways and genetic markers.

In contrast, a plausible explanation would distill this information into a form that is understandable for both clinicians and patients. The diversity of stakeholders in biomedicine, ranging from researchers and clinicians to patients and regulatory bodies, necessitates varying levels of explanation and communication. Researchers may require highly technical, accurate explanations that focus on algorithms and data structures, whereas clinicians may benefit more from practical, plausible explanations that emphasize clinical applicability [56]. In high-stakes scenarios like emergency medicine or critical care, where rapid decision-making is crucial, explanations must be quick to understand (plausible) and reliable (faithful). For example, in using ML models for predicting sepsis, clinicians need rapid, actionable insights that do not compromise the depth and accuracy of the information [68]. The dynamic nature of clinical settings calls for adaptive explanation models that can evolve through end-user feedback. This iterative process is essential for refining the balance between faithfulness and plausibility, helping fine-tune explanations for diverse applications, from routine diagnostics to personalized treatment planning.

Additionally, there is an educational aspect to consider. Providing explanations in biomedicine can educate clinicians about AI and ML, enhancing their ability to interact effectively with these technologies. This education can lead to a better understanding of what constitutes a ’good’ explanation in different contexts, further informing the balance between faithfulness and plausibility. Leveraging advanced visualization tools and interactive interfaces could make complex, faithful explanations more accessible and understandable, thus enhancing their plausibility without compromising depth. In conclusion, effectively navigating the faithfulness-plausibility trade-off requires a multifaceted approach that considers the complexity of biomedical data, the diverse needs of stakeholders, the critical nature of decision-making in medicine, and the potential for education and technological enhancement. This balancing act is crucial for ensuring that ML models are both technically sound and practically useful, and trustworthy in a biomedical context.

## Related and future work

ML has undergone remarkable transformations over the last decade, with explainability emerging as a critical frontier. The drive towards explainability in ML is fueled by the need to understand, trust, and effectively manage the increasingly complex models at the heart of today’s AI applications. This section explores the evolution and open challenges of ML explainability, highlighting significant achievements and their comparison to broader advancements in ML.

### Evolution and achievements in machine learning explainability

The journey towards explainable ML began as a response to the black-box nature of many advanced ML models, especially deep neural networks. Early efforts in ML were primarily focused on enhancing performance metrics, with little emphasis on understanding the decision-making processes of models. However, the demand for explainability intensified as ML applications permeated sensitive and critical domains such as healthcare, finance, and autonomous systems. This shift began a concentrated effort to develop methodologies and tools that could provide insights into how complex models make decisions. One of the first major milestones in the evolution of XAI was the development and popularization of feature-importance methods, such as LIME and SHAP. These methods aim to explain the predictions of any ML model by approximating the model locally around the prediction and attributing the contribution of each feature to the final decision. LIME, introduced by Ribeiro et al., and SHAP, introduced by Lundberg and Lee, have become foundational tools in the XAI toolkit, enabling users to gain insights into the predictive mechanisms of complex models [9, 10]. Techniques such as partial dependence plots (PDP) and individual conditional expectation (ICE) plots have also been instrumental in visualizing the relationship between features and the prediction outcome, further enhancing our understanding of model behavior.

In the realm of DL, the introduction of techniques like Gradient-weighted Class Activation Mapping (Grad-CAM) has provided a means to visually explain the decisions of convolutional neural networks, especially in image classification tasks. Grad-CAM, proposed by Selvaraju et al., utilizes the gradients of any target concept flowing into the final convolutional layer to produce a heatmap that highlights the important regions in the image for predicting the concept [26]. This method and its variants have been pivotal in making DL models more explainable to humans. Comparatively, while ML has achieved exponential growth in model sophistication and performance, the progress in explainability has been more measured. The achievements in XAI have provided tools to explain complex models and spurred a broader discussion on AI’s ethical and legal implications, leading to the development of guidelines and frameworks for responsible AI use. Notably, the European Union’s General Data Protection Regulation (GDPR) has significantly emphasized the importance of explainability in AI systems, influencing global perspectives on AI ethics and governance. Despite these advancements, the field of XAI remains in its early stages compared to the overall achievements in ML. The complexity of modern ML models, especially in DL, presents a continuous challenge for explainability. Techniques that offer deep insights into simpler models do not necessarily scale to more complex architectures, highlighting a gap between the capacity for explanation and the sophistication of models.

To summarize, the evolution of ML explainability over the last decade has been marked by significant achievements, from the development of local explanation methods to the advancement of model-agnostic explainability techniques and the visualization of DL decisions. These accomplishments have enhanced our understanding of complex ML models and established explainability as a cornerstone of ethical and responsible AI development. The ongoing dialogue between performance optimization and explainability underscores the dynamic and evolving nature of the field, pointing towards a future where AI’s power is matched by its transparency.

### Open gaps and challenges in machine learning explainability

Despite the significant strides made in enhancing the explainability of ML models, the field continues to face profound challenges and open gaps. These challenges stem from the intrinsic complexity of ML models, the diverse contexts in which they are applied, and the evolving nature of what constitutes sufficient explanation. Addressing these gaps is crucial for advancing trustworthy and responsible AI systems. One of the fundamental challenges in ML explainability is the trade-off between model complexity and explainability. As models become more complex, achieving high-performance levels on tasks ranging from natural language processing to image recognition, their decision-making processes become more difficult to explain. This complexity-explainability trade-off poses a dilemma: simplifying models to enhance explainability often means sacrificing some degree of performance, while optimizing for performance can lead to models that are nearly impossible to explain meaningfully. Bridging this gap requires innovative approaches to maintain or enhance performance without compromising explainability. Another significant challenge lies in the subjective nature of what is considered an adequate explanation. Different stakeholders, including data scientists, domain experts, and end-users, may require explanations tailored to their expertise and the application context. For instance, a data scientist might be interested in the technical details of feature importance. At the same time, an end-user may need explanations in plain language that relate to their personal experience or decision. Crafting universally satisfactory and comprehensible explanations across this spectrum of users remains an elusive goal.

Moreover, the dynamic and evolving landscapes of both technology and regulatory requirements add complexity to the challenge of explainability. As new models and techniques are developed, ensuring they adhere to the latest standards of explainability and ethical considerations requires continuous effort. Additionally, regulations such as the GDPR introduce legal imperatives for explainability, requiring methods to provide explanations that comply with such legal frameworks. Navigating these requirements while pushing the boundaries of ML innovation is a delicate balancing act. The scalability of explainability methods also presents a considerable challenge. While effective for smaller or less complex models, many existing techniques struggle to provide meaningful insights when applied to the scale and complexity of state-of-the-art DL models. This scalability issue is compounded by the fact that explanations must often be generated in real-time or near real-time to be useful in practical applications, demanding highly efficient and computationally feasible methods for explainability. Ultimately, there remains a pressing need for quantitative metrics to assess the effectiveness of explanations. While qualitative assessments can provide insights into the utility of an explanation, developing standardized, quantitative metrics that can objectively measure the quality of explanations is crucial. Such metrics would enable more rigorous benchmarking of explainability methods and facilitate progress in the field by providing clear targets for improvement.

In conclusion, the path toward fully explainable ML is fraught with technical, conceptual, and regulatory challenges. Bridging the gap between complex model architectures and the need for meaningful explanations requires a multifaceted approach. It necessitates ongoing research into new methods and technologies, a deep understanding of the diverse needs of stakeholders, and a commitment to navigating the evolving landscape of ethical and legal considerations. Addressing these challenges is not just a technical endeavor but a societal imperative, ensuring that the benefits of AI can be realized in a manner that is transparent, trustworthy, and aligned with human values.

The novel framework proposed in this work makes it clear that the XAI community must still face many challenges before claiming the explainability of a model. First, we observe an abundance of evidence for explaining black-box models. Yet, generating faithful interpretations is probably as hard as it is simple to be deceived by plausible yet untrue interpretations. Secondly, even if an interpretation might be correct (i.e., faithful), it still needs to be presented in a way that makes it easy for stakeholders to digest. These implications suggest exploring explainability from a novel perspective. Explainability should be an integral part of the software design and engage stakeholders at the earliest stages of developing an AI-based tool. Future research must address the problem of generating faithful interpretations, possibly through a top-down model design that accounts for both explainability and accuracy. Moreover, user studies should be integrated into the explainability design to understand how to deliver faithful, possibly plausible, and certainly human-understandable explanations. Going a step further, multiple explanation designs should be tested for their effectiveness in enabling the users to perform their tasks in an informed manner, which is the ultimate goal of explainability.

## Conclusions

In this work, by introducing formal terminology, we propose a novel theoretical framework that brings order and opportunities for a better design of explanations to the XAI community. The framework allows for dissecting explanations into evidence (factual data derived from the model) and interpretation (a hypothesized function that describes how the model utilizes the evidence). The explanation is the product of applying the interpretation to the evidence and is presented to the target user via an explanation interface.

These components enable the design of more principled explanations by defining the atomic components and the properties that enable them. There are three core properties: *(i)* the explanatory potential for the evidence (i.e., how much of the model the evidence can tell about); *(ii)* the faithfulness of the interpretation (i.e., whether the interpretation is true to the decision-making of the model); *(iii)* the plausibility of the explanation interface (i.e., how much the explanation makes sense to the user and is intelligible). We demonstrate that the theoretical framework can be applied to explanations from various methods that align with the proposed atomic components.

The lesson learned from analyzing explanations within the context of our proposed framework is that humans (both stakeholders and researchers) should be involved in the design of explainability as soon as possible in the AI-powered software design process, especially in sensitive application domains like biomedicine, where a blind application of black-box approaches hampers the right to an explanation. Involving stakeholders allows for the proper filling of each component in the theoretical framework of explainability and informs model design.

The top-down approach established in this way propels human understanding of how AI (and ML in particular) works, possibly fostering user trust in the system. We believe that high-stakes decision-making domains, such as biomedicine, would benefit the most from a more rigorous definition of core concepts of explainability, with opportunities to establish a conscious aid for AI-assisted decisions. Therefore, in future work, we want to apply our theoretical study to a real-case scenario in the biomedical sector and analyze its implementation with the help of human feedback to better focus on the plausibility analysis of our theoretical framework.

## Data Availability

Not applicable.
